# Reliable DNA Barcoding Performance Proved for Species and Island Populations of Comoran Squamate Reptiles

**DOI:** 10.1371/journal.pone.0073368

**Published:** 2013-09-12

**Authors:** Oliver Hawlitschek, Zoltán T. Nagy, Johannes Berger, Frank Glaw

**Affiliations:** 1 Zoologische Staatssammlung München, Munich, Germany; 2 Royal Belgian Institute of Natural Sciences, JEMU, Brussels, Belgium; State Natural History Museum, Germany

## Abstract

In the past decade, DNA barcoding became increasingly common as a method for species identification in biodiversity inventories and related studies. However, mainly due to technical obstacles, squamate reptiles have been the target of few barcoding studies. In this article, we present the results of a DNA barcoding study of squamates of the Comoros archipelago, a poorly studied group of oceanic islands close to and mostly colonized from Madagascar. The barcoding dataset presented here includes 27 of the 29 currently recognized squamate species of the Comoros, including 17 of the 18 endemic species. Some species considered endemic to the Comoros according to current taxonomy were found to cluster with non-Comoran lineages, probably due to poorly resolved taxonomy. All other species for which more than one barcode was obtained corresponded to distinct clusters useful for species identification by barcoding. In most species, even island populations could be distinguished using barcoding. Two cryptic species were identified using the DNA barcoding approach. The obtained barcoding topology, a Bayesian tree based on COI sequences of 5 genera, was compared with available multigene topologies, and in 3 cases, major incongruences between the two topologies became evident. Three of the multigene studies were initiated after initial screening of a preliminary version of the barcoding dataset presented here. We conclude that in the case of the squamates of the Comoros Islands, DNA barcoding has proven a very useful and efficient way of detecting isolated populations and promising starting points for subsequent research.

## Introduction

Since the pioneer studies of Hebert et al. [1], DNA barcoding has gained great popularity among biologists as a standardized, quick, and technically easy approach that does not require expert knowledge once reliable databases have been established. DNA barcoding has been applied in a broad range of studies and is helpful at various ends, such as biodiversity inventories of unstudied regions [2], [3], species identification through barcode databases [4], [5], pest identification and control [6], control of invasive species [7], [8], and human health [9]. One of the most common applications in biodiversity research is the use of DNA barcoding for a preliminary biodiversity assessment of a certain organism group in a certain region. This may range from very narrowly circumscribed target groups (e.g., [10]) to a broad range of organisms in large areas [11]–[13]. Despite its various uses and applications, DNA barcoding data were shown to have limited value to elucidate phylogenetic relationships [14] and sometimes 'disguise' species that cannot be identified by barcoding [15], [16].

In animals, the cytochrome *c* oxidase subunit I (COI) was established as the universal barcoding marker [1], mostly using the universal primers LCO and HCO [17]. However, COI is not equally easy to amplify in all taxonomic groups of animals. Until recently, non-avian reptiles were among the animal groups that were hard to barcode, and few studies focused on their COI DNA barcoding [18], [19]. Nagy et al. [20] published a barcoding study of the squamates and turtles of Madagascar. This was the first large-scale barcoding attempt targeting this group of vertebrates. The study focused on testing the efficiency of new primers for non-avian reptile barcoding, on detecting cryptic diversity, and on providing a barcode database for the easier identification of Malagasy species.

Like many other studies on Malagasy organisms, the DNA barcoding study [20] did not include the fauna of the Comoros archipelago. This group of four volcanic and hence fully oceanic islands is situated in the Western Indian Ocean halfway between the East African coast and Northwest Madagascar. Because of prevalent oceanic currents and winds, much of the Comoran biota originates from Madagascar, but is rich in endemic species [21], [22]. Nevertheless, only relatively few modern studies focused on Comoran organisms. Recent works on the phylogeny, biogeography and taxonomy of Comoran squamates were published by the group of S. Rocha [23]–[30] and by our group [22], [31], [32].

These studies showed that many endemic species of Comoran reptiles are highly threatened. Following the destruction of natural habitats, invasive species exotic to the archipelago were identified as one of the main factors of threat. Also, relatively high numbers of specimens were exported for the pet trade from some islands (reviewed in Hawlitschek et al. [22]). Molecular genetic studies might help identifying samples of animals by non-experts, e.g., for the control of the pet trade, for detecting inter-island transfer of endemic species, and for detecting newly introduced exotic species. These tasks can be difficult if relying on morphological characters only (e.g., in the case of *Hemidactylus* geckos [23]) and DNA barcoding is likely the most efficient method to cope with these problems.

In our work with Comoran squamates, we used a preliminary genetic screening, including DNA barcoding, to receive a preview on genetic divergences between species and island populations, to distinguish whether species were more likely native or introduced, and to detect possible cryptic species. Then, we used multigene approaches to study groups of squamates that were found to be interesting by our initial screening. In this article, we present the results of our DNA barcoding approach and, wherever possible, compare them with the results of available multigene phylogenies. We also tested the performance of DNA barcoding to correctly identify island populations of native species.

## Materials and Methods

### Sampling, permits, and ethics statements

No experiments were conducted using living animals. Furthermore, none of the samples were specifically collected for this project, but for an earlier study on Comoran reptiles [22] by 3 of the 4 authors of this paper (OH, JB, FG). We exclusively used museum samples which were already available and were deposited in a tissue bank at the Zoologische Staatssammlung München (ZSM), Germany. For all species and 176 out of 217 specimens, not only tissue samples but also voucher specimens were available (Tables 1 and S1). All samples and voucher specimens were analysed with permission of the ZSM. Voucher specimens were euthanized using approved methods (e.g. anaesthesia with ketamine, followed by ketamine overdose) that do not require approval by an ethics committee according to national law on the Comoros.

**Table 1:** Samples used in the DNA barcoding analysis.

Family/Species	Samples	Vouchers	RepCOI	LCO/HCO	Total sequences	Success
**Agamidae**	2	2	2	0	2	100%
*Agama agama*	2	2	2	0	2	100%
**Iguanidae**	1	1	1	0	1	100%
*Oplurus cuvieri*	1	1	1	0	1	100%
**Chamaeleonidae**	10	7	10	4(4)	10	100%
*Furcifer cephalolepis*	6	4	6	2(2)	6	100%
*Furcifer polleni*	4	3	4	2(2)	4	100%
**Typhlopidae**	29	29	0	24	24	83%
*Ramphotyphlops braminus*	26	26	0	22	22	85%
*Typhlops comorensis*	2	2	0	2	2	100%
*Typhlops* sp.	1	1	0	0	0	0%
**Lamprophiidae**	14	14	14	2	14	100%
*Liophidium mayottensis*	1	1	1	0	1	100%
*Lycodryas cococola*	8	8	8	1	8	100%
*Lycodryas maculatus*	5	5	5	1	5	100%
**Scincidae**	52	52	26	9	26	50%
*Amphiglossus johannae*	25	25	18	9(9)	18	72%
*Crytoblepharus boutonii*	8	8	7	0(6)	7	88%
*Trachylepis comorensis*	18	18	1	0	1	6%
*Trachylepis striata*	1	1	0	0	0	0%
**Gekkonidae**	108	81	91	38	91	84%
*Ebenavia inunguis*	7	7	6	1	6	86%
*Geckolepis maculata*	12	12	10	9	10	77%
*Hemidactylus frenatus*	7	6	7	4	7	100%
*Hemidactylus mercatorius*	9	7	9	0	9	100%
*Hemidactylus parvimaculatus*	4	4	1	0	1	25%
*Hemidactylus platycephalus*	14	14	14	0	14	100%
*Paroedura sanctijohannis*	12	12	9	0	9	75%
*Paroedura stellata*	6	6	6	0	6	100%
*Phelsuma comorensis*	3	1	2	2(2)	2	67%
*Phelsuma dubia*	8	2	8	7(7)	8	100%
*Phelsuma laticauda*	8	1	7	5(7)	7	88%
*Phelsuma nigristriata*	2	1	2	1(1)	2	100%
*Phelsuma pasteuri*	2	2	1	0(0)	1	50%
*Phelsuma robertmertensi*	3	1	2	2(2)	2	67%
*Phelsuma v-nigra*	12	3	7	7	7	58%

Sequences of non-Comoran species (mostly from Madgascar; all taken from GenBank, with the exception of *Amphiglossus ardouini*) are not listed. The values given for families are sums of all species comprised. Samples: the total number of samples that were attempted to sequence. Vouchers: the number of samples for which a voucher specimen is available. RepCOI: the number of sequences that were obtained using the primer pair RepCOI-F/RepCOI-R [20]. LCO/HCO: the number of sequences that were obtained using the primer pair LCO/HCO [17]; the number of brackets lists the number of samples attempted to amplify with HCO/LCO, if different from the number given in "Samples". Total sequences: the total number of sequences obtained.

Collection and transport of specimens was conducted with the following permits: (1) Issued by the Direction Générale de l'Environnement, Moroni, Union des Comores: research and export permit (no permit number, 1st March 2000), research permit (02/121/MPE/DGE, 12th April 2002), export permit (02/141/MPE/DGE, 2002), research and export permit (no permit number, 12th March 2008), research permit (CNDRS/08/2010, 22nd January 2010), export permit (CNDRS/030/2010, 5th April 2010). (2) Issued by the Direction de l'Agriculture et de la Forêt, Mayotte, France: research and export permit (no permit number, 23rd February 2000), research and export permit (24/DAF/SEF/2008, 19th March 2008), research and export permit (2010-13/DAF/SEF, 30th March 2010). Import of species protected by CITES into Germany was approved by the German authorities (Bundesamt für Naturschutz, Bonn).

### Laboratory protocols

Total genomic DNA was extracted using the standard protocols of the NucleoSpin® 96 Tissue kit (Macherey-Nagel) and the DNEasy Tissue Kit (Qiagen, Hilden, Germany). We amplified the 5’ half of COI using the primers RepCOI-F/RepCOI-R [20] or LCO/HCO [17] and the corresponding PCR protocols. Table 1 lists which primer combination was more successful for each species. Sequencing was conducted using the BigDye® Terminator v1.1 Cycle Sequencing Kit on ABI 3730 and ABI 3130xl capillary sequencers (Life Technologies). Sequence data were deposited in BOLD and GenBank and are available under accession numbers KF604749 to KF604886 (Table S1).

### Barcoding tree reconstruction

We used Sequencher 4.9 © for editing and quality checking of the chromatograms, Mesquite 2.72 [33] for additional quality checking, including inspection of protein translations, and MAFFT 6 [34], [35] for alignment of the COI dataset. In addition to the sequences produced from Comoran samples, we added 34 sequences from related species obtained from GenBank (most originate from [20]) for comparison with the barcoding dataset. We selected sequences that were found to be most similar to the Comoran sequences in BLAST searches. We then conducted a test of substitution saturation [36], [37] in DAMBE v5.2.34 [38] and plotted transitions and transversions against Kimura 2-parameter (K2p) divergences to visualize possible saturation at a higher divergence level.

We calculated pairwise K2p-distances in MEGA 5.0 [39]. We partitioned the dataset according to codon position and in order to identify appropriate substitution models for the maximum likelihood (ML) and Bayesian analyses, we used jModeltest 0.1.1 [40]. We assessed AIC and BIC results, giving BIC preference over AIC. Subsequently, we conducted (1) ML analyses with 1,000 fast bootstrap repeats in raxmlGUI 1.0 [41], [42] and (2) Bayesian analyses in MrBayes 3.1.2 [43] on the CIPRES portal 2.2 [44] with two runs and four chains with 30,000,000 generations (samplefreq = 1,000, 25% burnin). MrBayes runs were checked for convergence and normal distribution in Tracer v1.5 [45].

One aim of this analysis was to test the performance of DNA barcoding versus multigene phylogenies, wherever available. We used data for the genera *Cryptoblepharus* (766 bp) [34], *Ebenavia* (1894 bp; unpublished data by O. Hawlitschek), *Lycodryas* (3498 bp) [31], *Phelsuma* (2872 bp) [27], and *Paroedura* (3174 bp) [32]. Then, we estimated trees using MrBayes with the setting described above, but only run for 10,000,000 generations. The subsets of our barcoding dataset and the corresponding multigene datasets contained only the genus in question and related taxa.

### Clustering and species identification by barcoding

To measure the success of the identification of species and island populations of native species in our dataset using DNA barcodes we used an objective clustering approach as implemented in SpeciesIdentifier [46]. This software clusters sequences using p*-*distances, thus allowing the comparison of clusters with the existing taxonomy [10], [47]). Species names and clustering thresholds are preset by the user. We conducted clustering analyses with thresholds of 5% to 15% for delimitation of 'barcoding species', and 0.2% to 2.0% for delimitation of island populations. Additionally, we conducted query identification analyses of the dataset with the 'best match' and 'best close match' criteria [46]. Under the 'best match' criterion, any query sequence is assigned the species name of its best matching barcode (i.e., reference sequence). If this analysis is run in SpeciesIdentifier, the output shows how many sequences were assigned to a matching sequence in agreement with their pre-assigned species name. Obviously, the sequences of species of which only a single sequence is included in the dataset are automatically misidentified because their best matching sequence belongs to a different species. Applying the 'best close match' criterion, the same analysis is refined with a user-defined cutoff distance. Sequences that do not match within the defined cutoff distance are not assigned to the barcoding species of their best matching sequence, but to a barcoding species of their own. For the clustering analyses for species identification, we used a dataset from which all identical haplotypes were cropped using the software Collapse 1.2 [48]. All sequences from non-Comoran species were removed manually. The cropped dataset consisted of 130 sequences. In the dataset for the identification of island populations, we additionally removed all species that were considered non-native [22], leaving 61 sequences.

## Results and Discussion

### The DNA barcoding dataset

We produced a total of 168 DNA barcodes for 27 out of the 29 currently recognized species of Comoran squamates (Table 1) including 2 recently described species, *Lycodryas cococola* [31] and *Paroedura stellata* [32]. We also included all recognized subspecies of Comoran species, including *Cryptoblepharus boutonii ater* (Grand Comoro, corresponding to *C. ater* according to Horner [49]), *C. b. degrijsii* ( Anjouan, corresponding to *C. quinquetaeniatus*), *C. b. mayottensis* (Mayotte, corresponding to *C. gloriosus mayottensis*), *C. b. mohelicus* (Mohéli, corresponding to *C. g. mohelicus*), *Lycodryas cococola cococola* (Grand Comoro), *L. c. innocens* (Mohéli), *L. maculatus maculatus* (Anjouan), *L. m. comorensis* (Mayotte), *Phelsuma v-nigra v-nigra* (Mohéli), *P. v. anjouanensis* (Anjouan), and *P. v. comoraegrandensis* (Grand Comoro).

A single barcode sequence was obtained for 5 species, 2 or more DNA barcodes for all other species, with an overall high success rate of 100% in 12 species and >70% in further 7 species (excluding the species for which a single sample was available and successfully sequenced). The highest success rates in PCR amplification and sequencing were achieved using the primers RepCOI-F and RepCOI-R [20]. However, for a number of species LCO and HCO [17] worked better (Table 1). Notably, both primer pairs failed to produce readable sequences in the most common Comoran reptile species, *Trachylepis comorensis*. Only a single sequence could be produced for this species, based on a sample of an egg; all of the numerous samples of muscle tissue failed. Neither could any sequence be produced for the related, non-native *T. striata*. Furthermore, a sample of an undescribed species of *Typhlops* could not be sequenced. *Ramphotyphlops braminus* and *Typhlops comorensis* were the only species in which no sequence was produced with RepCOI-F/RepCOI-R, but LCO/HCO performed well.

K2p-distances are given in Table 2 for families and in Table 3 for species or clades endemic to the Comoros. Within many species inhabiting more than one island of the archipelago, genetic divergences range from 4.8% to 9.4% (K2p distance), with an average around 3%. Notably, this comprises endemic clades whose lineages can be clearly attributed to islands (*Phelsuma v-nigra*, *Geckolepis maculata*, *Cryptoblepharus boutonii*), as well as non-endemic groups whose lineages are mixed between the islands (*Hemidactylus* spp.). Other introduced taxa (*Phelsuma laticauda*, *P. dubia*, *Ramphotyphlops braminus*) show much lower divergences from 0.02% to 1.3%. *R. braminus* is the only all-female snake known to reproduce parthenogenetically, which means that a single specimen can found a population with its clonally produced offspring and may explain the exceptionally low haplotype diversity [50].

**Table 2:** Genetic divergences within families of Comoran squamates.

Family	Avg. distance between Comoran species	Avg. distance between Malagasy species (Nagy et al. [20])
Chamaeleonidae	12.5 (11.6–13.9)	23.7
Typhlopidae	22.5 (22.0–23.0)	18.6
Lamprophiidae	14.1 (10.4–20.3)	20.2
Scincidae	26.8 (23.5–29.4)	22.2
Gekkonidae	28.5 (11.9–35.7)	29.8

All genetic divergences are given as K2p-distances. Agamidae and Iguanidae are each represented by a single species only and are not shown.

**Table 3:** Maximum genetic divergences between and within island populations of Comoran squamates.

Species	Max. overall K2p distance	Anjouan	Grand Comoro	Mayotte	Mohéli
*Furcifer cephalolepis*	2.8	-	2.8 (N = 6)	-	-
*Furcifer polleni*	1.1	0 (N = 1)	-	1.1 (N = 3)	-
*Ramphotyphlops braminus*	0.2	0 (N = 7)	0.2 (N = 2)	0 (N = 3)	0 (N = 10)
*Typhlops comorensis*	8.1	0 (N = 1)	0 (N = 1)	-	-
*Lycodryas cococola*	6.6	-	0.8 (N = 3)	-	0.5 (N = 5)
*Lycodryas maculatus*	6.4	0.3 (N = 3)	-	0.2 (N = 2)	-
*Amphiglossus johannae*	6.8	0.5 (N = 5)	0.2 (N = 2)	0.5 (N = 2)	1.4 (N = 9)
*Cryptoblepharus boutonii*	5.3	1.9 (N = 3)	0.2 (N = 2)	0 (N = 1)	0 (N = 1)
*Ebenavia inunguis*	22.0	0 (N = 1)	0.4 (N = 2)	0 (N = 1)	0 (N = 2)
*Geckolepis maculata*	4.8	1.3 (N = 5)	0 (N = 2)	0.7 (N = 2)	0 (N = 1)
*Hemidactylus frenatus*	6.7	0.2 (N = 3)	0 (N = 1)	0 (N = 1)	0 (N = 2)
*Hemidactylus mercatorius*	6.5	2.5 (N = 6)	0 (N = 2)	0 (N = 1)	0 (N = 0)
*Hemidactylus platycephalus*	5.5	5.3 (N = 8)	1.1 (N = 3)	0 (N = 1)	5.5 (N = 2)
*Paroedura sanctijohannis*	8.2	0.4 (N = 6)	4.1 (N = 2)	-	0 (N = 1)
*Paroedura stellata*	1.3	-	-	1.3 (N = 6)	-
*Phelsuma comorensis*	0	-	0 (N = 2)	-	-
*Phelsuma dubia*	1.3	0.7 (N = 3)	0.4 (N = 2)	0.9 (N = 2)	0 (N = 1)
*Phelsuma laticauda*	0.2	0 (N = 6)	-	0 (N = 1)	-
*Phelsuma nigristriata*	0.2	-	-	0.2 (N = 2)	-
*Phelsuma robertmertensi*	0.9	-	-	0.9 (N = 2)	-
*Phelsuma v-nigra*	9.4	4.3 (N = 2)	0.4 (N = 3)	-	0 (N = 2)
Comoran *Phelsuma* radiation*	24.4	4.3 (N = 2)	0.4 (N = 3)	12.3 (N = 3)	0 (N = 2)
Comoran *Lycodryas* radiation**	10.4	0.3 (N = 3)	0.8 (N = 3)	0.2 (N = 2)	0.5 (N = 5)

All genetic divergences are given as % of K2p-distances. Species for which only a single sequence is available are not included. * This includes *P. v-nigra*, *P. pasteuri* and *P. robertmertensi*. ** This includes *L. cococola* and *L. maculatus*.

In the analysis of substitution saturation in DAMBE, the index of substitution saturation Iss was always significantly below its critical value Iss.c. This indicates an overall low saturation in the dataset. The plotting of transitions and transversions against divergence indicated saturation at higher levels of divergence (results not shown).

### Clustering, identification of species and island populations

The objective clustering analysis for species identification under thresholds from 5% to 15% yielded a varying number of clusters, ranging from 25 to 37. The number of clusters never corresponded exactly to the number of taxonomic species included (27, pre-defined according to current taxonomy). The best results were achieved under thresholds of 8% to 11% with a total of 28 clusters, 24 of which were in correspondence to the currently valid taxonomy. Because of the high divergences between the island populations of *Ebenavia inunguis*, these samples did not form a common cluster at thresholds that yielded appropriate results for other species. At the same level, however, the 2 Comoran species of *Lycodryas* formed a common cluster.

The 'best match' query analysis correctly linked 124 out of 130 barcoding sequences to taxonomic species. The remaining 6 sequences refer to species that are represented by a single sequence only in the clustering dataset, and are thus automatically misidentified by the 'best match' analysis. The 'best closest match' query analysis correctly linked 123 sequences at thresholds of 8% to 11%. This supported the view that all Comoran squamate species included in this study are monophyletic, if sequences of non-Comoran origin are excluded.

The objective clustering analyses for the identification of island populations of native species yielded 27 to 48 clusters. Thus, the number of clusters corresponded to the 27 island populations of 9 included species at thresholds from 1.6% to 2.0%. However, the highest number of clusters corresponding directly to actual island populations was 24 at a clustering threshold of 1.2%. At higher thresholds, island populations were lumped. The 'best match' query linked 50 out of 61 barcoding sequences to the correct island populations. The 'best closest match' query correctly linked 47 sequences at thresholds of 1.4% or higher.

We want to stress that the results of these clustering analyses should be seen specifically for this dataset. As discussed by many authors, clustering thresholds for the delimitation of species and populations vary widely across organisms [51]. In our analyses, the combination of all objective clustering criteria not only allows the identification of barcodes to species level, but also to the level of island populations, with good performance as long as native species with monophyletic island populations are concerned.

### Topologies constructed in DNA barcoding vs. multigene phylogenies

**Figure 1:**
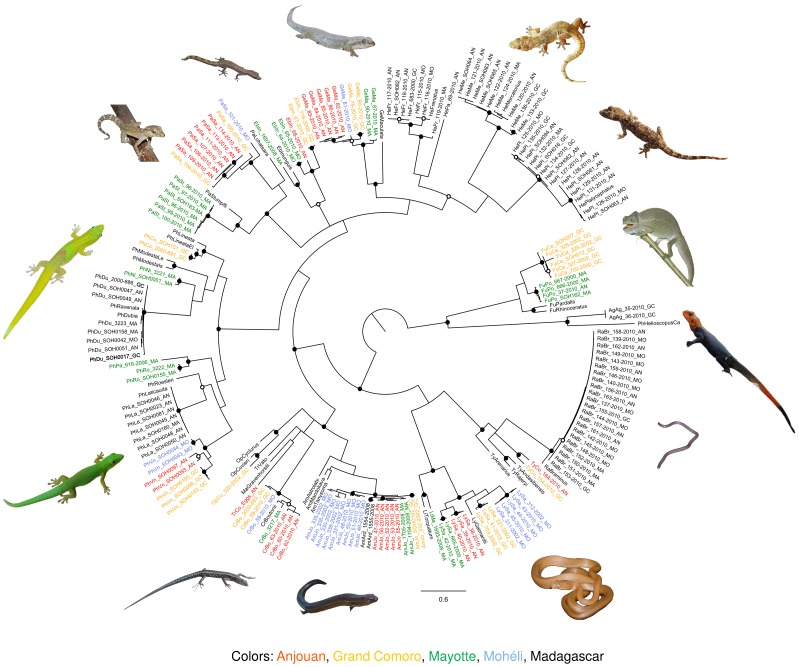
Bayesian tree of the COI dataset. Nodes with Bayesian PP and ML bootstrap support ≥ 90% are marked with filled black circles, nodes with Bayesian PP or ML bootstrap support ≥ 90% are marked with empty black circles. Island lineages of endemic species are marked in colors.

The barcoding topology based on a Bayesian tree is shown in Fig. 1. All genera, including Comoran species and selected related species, were retrieved monophyletic. As in the clustering analysis, all species were retrieved monophyletic, with the exceptions of *Phelsuma dubia* and *Amphiglossus johannae*. In our trees, a sequence of the Malagasy *P. ravenala* is nested within the branch comprising Comoran samples of *P. dubia*. In *Amphiglossus*, the sequences of the Malagasy *A. ardouini* are nested within the Comoran endemic *A. johannae*.

**Figure 2:**
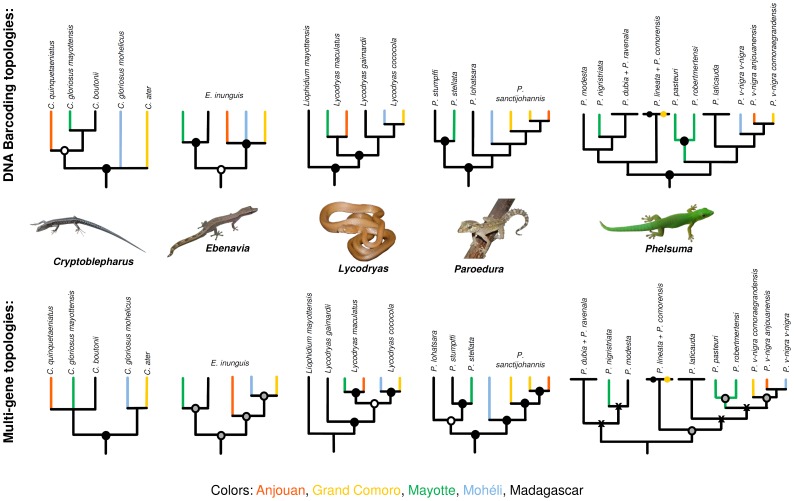
A comparison of topologies from our DNA barcoding analyses with topologies from multigene analyses for 5 genera of squamates with endemic Comoran lineages. Nodes with at least two values out of Bayesian PP, ML bootstrap, or Parsimony bootstrap support ≥ 90% are marked with filled black circles, nodes with at least one value out of Bayesian PP, ML bootstrap, or Parsimony bootstrap support ≥ 90% are marked with empty black circles. If only a single support value is available for the phylogeny, black circles filled with grey mark nodes with support values of ≥ 90%, and 'X' mark nodes with support values of ≥ 80%. The topologies were cropped to highlight lineages that are endemic to a single island, marked by color. Lineages that are present in the Comoros, but not endemic, are not highlighted. The multigene topologies are taken from the following studies: *Cryptoblepharus* [25], *Ebenavia* (unpublished data by O. Hawlitschek), *Lycodryas* [31], *Paroedura* [31], *Phelsuma* [27].

Fig. 2 shows 5 subsets of the barcoding topology. The subsets were cropped so that only representatives of major clades are displayed. A comparison of the trees with topologies of multigene phylogenies [25]–[27], [31], [32] shows major incongruences between the topologies in 3 of these 5 cases. However, these incongruences are often poorly supported, and the support values for the nodes concerned in the barcoding topology are generally poor.

Despite these incongruences, the DNA barcoding tree yielded overall very similar results to the multigene trees. We maintain that DNA barcoding is not an adequate method for species delimitation or phylogenetic reconstruction if used alone. However, the comparison of multigene analyses with analyses based on the barcoding dataset only demonstrated the ability of the DNA barcoding approach to provide a raw preview of phylogenetic relationships and to lay incentives for further studies.

### Patterns of genetic divergence in island populations

As shown in Figs. 1 and 2, and Tables 4 and 5, DNA barcodes of Comoran squamates are in most cases not only useful to identify which species a sample belongs to, but also in which island population it was collected. This was possible for all included samples of the endemics *Lycodryas cococola*, *L. maculatus*, *Phelsuma v-nigra*, *Typhlops comorensis*, and the Comoran clades of *Cryptoblepharus boutonii*, *Ebenavia inunguis*, and *Geckolepis maculata*. In *E. inunguis*, the topology suggested that the Comoros are inhabited by 2 clades resulting from separate colonization events, as previously hypothesized for other endemic squamates [24], [26], [27], [32].

**Table 4:** Results of the objective clustering analyses of species.

Clustering threshold	No. clusters	No. clusters corresponding to taxonomy	Max. No. species per cluster	No. correct identifications by 'best closest match'
5%	37	20	1	116
6%	36	21	1	116
7%	34	23	1	117
8%	28	24	2	123
9%	28	24	2	123
10%	28	24	2	123
11%	28	24	2	123
12%	25	22	2	123*
13%	25	20	2	123*
14%	25	22	2	123*
15%	25	22	2	123*

Clustering was conducted in SpeciesIdentifier with arbitrary thresholds of 5% to 15%. The dataset used here contained 130 sequences belonging to 27 species. 6 species were represented by a single sequence. 124 sequences were correctly identified by the 'best match' criterion. *At these clustering thresholds, the 'best closest match' query criterion yielded 1 misidentification.

**Table 5:** Results of the objective clustering analyses of island populations.

Clustering threshold	No. clusters	No. clusters corresponding to island population	Max. No. populations per cluster	No. correct identifications by 'best closest match'
0.2%	48	11	1	23
0.4%	41	18	1	34
0.6%	39	20	1	37
0.8%	34	23	1	42
1.0%	32	23	1	45
1.2%	31	24	1	46
1.4%	29	23	2	47*
1.6%	27	20	2	47**
1.8%	27	20	2	47**
2.0%	27	20	2	47**

Clustering was conducted in SpeciesIdentifier with arbitrary thresholds of 0.2% to 2.0%. The dataset used here contained 61 sequences belonging to 27 island populations of 9 native species. 9 island populations were represented by a single sequence. 50 sequences were correctly identified by the 'best match' criterion. At higher clustering thresholds, the 'best closest match' query criterion yielded 1 (*) or 2 (*) misidentifications.

In other cases, the most recently diverged island lineages could not be distinguished, whereas the more distant island lineages were distinct. This was found in the endemic *Amphiglossus johannae* and *Paroedura sanctijohannis*. This pattern may be explained by fast speciation after the colonization of new islands, by the introgression of haplotypes, or by past extinction and re-colonization events that 'disguise' the orginal pattern of divergence [32]. Notably, the presumably introduced geckos *Hemidactylus frenatus* and *H. mercatorius* showed a similar pattern. However, because of the low sample sizes for some rarer species we cannot exclude that incomplete lineage sorting between island populations may be higher than shown by our results. This is also the reason why no statement can be made on some species. In some introduced species, haplotypes are mixed over all colonized islands, such as in *Hemidactylus platycephalus*, *Ramphotyphlops braminus*, *Phelsuma dubia* and *P. laticauda*, which supports the view that the Comoran populations of these anthropophilous species originated from recent introduction events.

Endemic taxa that are restricted to a single island – and hence probably resulting from isolated colonization events [27], [32] – also show relatively little genetic diversification of 0.2% to 1.3%. In contrast to this, the Grand Comoran endemic *Furcifer cephalolepis* shows high intraspecific divergences (up to 2.8%), which has also been shown for an independent set of samples and different molecular markers [24]. Grand Comoro’s endemic populations of other squamates also show higher genetic divergences than populations on other islands [24], [31], [32]. This is remarkable because Grand Comoro is assumed to be the geologically youngest major island of the archipelago [52]. As discussed in Hawlitschek & Glaw [32], reasons for this may be that either Grand Comoro is geologically older than currently estimated, or populations of geologically older islands are younger because these islands were colonized later in geological history, e.g., after the extinction of an earlier island population.

### The barcoding topology at species level and its significance for taxonomy

Of the 22 Comoran squamate species for which at least 2 barcodes were produced, 19 were retrieved as monophyletic units. We explore the cases of the species that were not retrieved monophyletic and examine the reason for this incongruence between DNA barcoding and existing taxonomy.

The Malagasy day gecko *Phelsuma ravenala*, described by Raxworthy et al. [53], is nested within *P. dubia* from Madagascar and the Comoros in our barcoding tree, with K2p distances of less than 1% from all included *P. dubia* sequences. This is congruent with the results of Rocha et al. [27]. In their original description, the authors presented the number of scale rows around midbody as an important character to distinguish between the 2 species. However, a morphological study of this character in Comoran *P. dubia* [54] showed that these specimens were outside the ranges given for *P. dubia* and *P. ravenala* by Raxworthy et al. [53], suggesting that the validity of the latter species is in need of confirmation.

The Comoran endemic *Phelsuma comorensis* is found nested within the otherwise Malagasy *P. lineata* in our barcoding tree. This is also congruent with the results of Rocha et al. [27]. The minimal K2p distance from *P. lineata* sequences is 3.1%. The polytypic *P. lineata* has been shown to be a species with variable morphology and ecological adaptability [30], and *P. comorensis* could be argued to fall within the ranges of these amplitudes.

*Amphiglossus johannae*, a skink considered endemic to the Comoros, is retrieved paraphyletic with respect to the Malagasy *A. ardouini*. While both species are easily distinguished via external morphological characters, the minimal K2p distance between them is 0.5%. The reason for this unexpected position of the 2 species in the barcoding tree is unknown. Future studies should explore the possibility that *A. johannae* represents a case of recent, but natural dispersal from Madagascar to the Comoros with rapid adaptation of the morphological characters to the insular environment (but see [30] for alternative scenarios).

The Comoran populations of the gecko genus *Paroedura* and the snake genus *Lycodryas*, formerly considered as *Paroedura sanctijohannis* and *Lycodryas sanctijohannis*, respectively, are retrieved paraphyletic in our barcoding tree. The paraphyly of Comoran *Paroedura* was confirmed by molecular and morphological data [32], whereas Comoran *Lycodryas* were found to be monophyletic [31]. *Lycodryas* is a good example of a case in which DNA barcoding results are incongruent with the current taxonomy, but are not confirmed by a multigene analysis. As discussed in Hawlitschek et al. [31], previous analyses based on few mtDNA markers also suggested this paraphyly, which stands in contrast to the results of morphological studies. Only a multigene analysis including a larger mtDNA dataset and nuclear DNA markers confirmed the monophyly of Comoran *Lycodryas*.

With the exception of the cases stated so far, all taxonomic species are represented by monophyletic and clearly distinct clusters. As described, most detected cases of species paraphyly can likely be attributed to a poorly resolved taxonomy of the species in question. This means that – once taxonomy is revised – all the 22 Comoran squamate species for which at least 2 barcodes were produced will be correctly identified by DNA barcoding.

## Supporting Information

Table S1 **A list of all samples included in the DNA barcoding study of Comoran squamates.** The list includes voucher specimens, collecting details, and accession numbers for GenBank and BOLD.
(XLS)

## References

[1] Hebert PDN, Cywinska A, Ball SL, deWaard JR (2003) Biological identifications through DNA barcodes. Proc R Soc B 270: 313–321. 10.1098/rspb.2002.2218 PMC1691236 12614582

[2] Hajibabaei M, Singer GAC, Hebert PDN, Hickey DA (2007) DNA barcoding: how it complements taxonomy, molecular phylogenetics and population genetics. Trends Genet 23: 167–172. 17316886 10.1016/j.tig.2007.02.001

[3] Janzen DH, Hallwachs W, Blandin P, Burns JM, Cadiou JM, et al (2009) Integration of DNA barcoding into an ongoing inventory of complex tropical biodiversity. Mol Ecol Res 9: 1–26. 10.1111/j.1755-0998.2009.02628.x 21564960

[4] Savolainen V, Cowan RS, Vogler AP, Roderick GK, Lane R (2005) Towards writing the encyclopedia of life: an introduction to DNA barcoding. Phil Trans R Soc B 360: 1805–1811. 16214739 10.1098/rstb.2005.1730 PMC1609222

[5] Ratnasingham S, Hebert PDN (2007) BOLD: The Barcode of Life Data System (www.barcodinglife.org). Mol Ecol Notes. 7: 355–364. 10.1111/j.1471-8286.2007.01678.x PMC1890991 18784790

[6] Ball SL, Armstrong KF (2006) DNA barcodes for insect pest identification: a test case with tussock moths (Lepidoptera: Lymantriidae). Canad J Forest R 36(2): 337–350.

[7] Armstrong KF, Ball SL (2005) DNA barcodes for biosecurity: invasive species identification. Philos Trans R Soc Lond B 360: 1813–1823. 16214740 10.1098/rstb.2005.1713 PMC1609225

[8] Scheffer SJ, Lewis ML, Joshi RC (2006) DNA barcoding applied to invasive leafminers (Diptera: Agromyzidae) in the Philippines. Ann Ent Soc Am 99(2): 204–210.

[9] Lowenstein JH, Burger J, Jeitner CW, Amato G, Kolokotronis SO, et al (2010) DNA barcodes reveal species-specific mercury levels in tuna sushi that pose a health risk to consumers. Biol Lett 6(5): 692–695. 20410032 10.1098/rsbl.2010.0156 PMC2936149

[10] Hendrich L, Pons J, Ribera I, Balke M (2010) Mitochondrial Cox1 sequence data reliably uncover patterns of insect diversity but suffer from high lineage-idiosyncratic error rates. PLoS ONE 5(12): e14448. 21203427 10.1371/journal.pone.0014448 PMC3010977

[11] Haszprunar G (2009) Barcoding Fauna Bavarica – eine Chance für die Entomologie. NachrBl bayer Ent 58(1/2): 45/47.

[12] Gonzalez MA, Baraloto C, Engel J, Mori SA, Pétronelli P, et al (2009) Identification of Amazonian trees with DNA barcodes. PLoS ONE 4(10): e7483. 19834612 10.1371/journal.pone.0007483 PMC2759516

[13] Hausmann A, Haszprunar G, Hebert PDN (2011) DNA Barcoding the Geometrid Fauna of Bavaria (Lepidoptera): Successes, Surprises, and Questions. PLoS ONE 6(2): e17134. 21423340 10.1371/journal.pone.0017134 PMC3040642

[14] Vences M, Thomas M, van der Meijden A, Chiari Y, Vieites DR (2005) Comparative performance of the 16S rRNA gene in DNA barcoding of amphibians. Frontiers Zool 2: 5. 10.1186/1742-9994-2-5 PMC555853 15771783

[15] Meyer CP, Paulay G (2005) DNA barcoding: Error rates based on comprehensive sampling. PLoS Biol 3(12): e422. 16336051 10.1371/journal.pbio.0030422 PMC1287506

[16] Hickerson MJ, Meyer CP, Moritz C (2006) DNA barcoding will often fail to discover new animal species over broad parameter space. Syst Biol 55: 729–739. 17060195 10.1080/10635150600969898

[17] Folmer O, Black M, Hoeh W, Lutz R, Vrijenhoek R (1994) DNA primers for amplification of mitochondrial cytochrome c oxidase subunit I from diverse metazoan invertebrates. Mol Mar Biol Biotech 3: 294–299. 7881515

[18] Vences M, Nagy ZT, Sonet G, Verheyen E (2012) DNA barcoding amphibians and reptiles. In: Kress WJ, Erickson DL, editors. DNA barcodes: Methods and protocols. Methods in Molecular Biology 858. pp. 79–107. 10.1007/978-1-61779-591-6_5 22684953

[19] Murphy RW, Crawford AJ, Bauer AM, Che J, Donnellan SC, et al (2013) Cold Code: the global initiative to DNA barcode amphibians and nonavian reptiles. Mol Ecol Res 13 161–167.

[20] Nagy ZT, Sonet G, Glaw F, Vences M (2012) First large-scale DNA barcoding assessment of reptiles in the biodiversity hotspot of Madagascar, based on newly designed COI primers. PLoS ONE 7(3): e34506. 22479636 10.1371/journal.pone.0034506 PMC3316696

[21] Louette M, Meirte D, Jocqué R (2004) La faune terrestre de'l archipel des Comores. Studies in Afrotropical Zoology, 293. Tervuren: MRAC. 456 pp.

[22] Hawlitschek O, Brückmann B, Berger J, Green K, Glaw F (2011) Integrating field surveys and remote sensing data to study distribution, habitat use and conservation status of the herpetofauna of the Comoro Islands. ZooKeys 144: 21–79. 10.3897/zookeys.144.1648 PMC3233692 22207785

[23] Rocha S, Carretero MA, Harris DJ (2005) Diversity and phylogenetic relationships of *Hemidactylus* geckos from the Comoro islands. Mol Phylogenet Evol 35: 292–299. 15737599 10.1016/j.ympev.2004.11.023

[24] Rocha S, Carretero MA, Harris DJ (2005) Mitochondrial DNA sequence data suggests two independent colonizations of the Comoros archipelago by chameleons of the genus *Furcifer* . Belg J Zool 135: 39–42.

[25] Rocha S, Carretero MA, Vences M, Glaw F, Harris DJ (2006) Deciphering patterns of transoceanic dispersal: the evolutionary origin and biogeography of coastal lizards (*Cryptoblepharus*) in the Western Indian Ocean region. J Biogeogr 33: 13–22.

[26] Rocha S, Posada D, Carretero MA, Harris DJ (2007) Phylogenetic affinities of Comoroan and East African day geckos (genus *Phelsuma*): Multiple natural colonisations, introductions and island radiations. Mol Phylogenet Evol 43: 685–692. 17113791 10.1016/j.ympev.2006.07.010

[27] Rocha S, Vences M, Glaw F, Posada D, Harris DJ (2009) Multigene phylogeny of Malagasy day geckos of the genus *Phelsuma* . Mol Phylogenet Evol 52: 530–537. 19362158 10.1016/j.ympev.2009.03.032

[28] Rocha S, Carretero MA, Harris DJ (2010) Genetic diversity and phylogenetic relationships of *Mabuya* spp. (Squamata: Scincidae) from western Indian Ocean islands. Amphibia-Reptilia 31: 375–385.

[29] Rocha S, Carretero MA, Harris DJ (2010) On the diversity, colonization patterns and status of *Hemidactylus* spp. (Reptilia: Gekkonidae) from the Western Indian Ocean islands. Herpetol J 20: 83–89.

[30] Rocha S, Rösler H, Gehring PS, Glaw F, Posada D, et al (2010) Phylogenetic systematics of day geckos, genus *Phelsuma*, based on molecular and morphological data (Squamata: Gekkonidae). Zootaxa 2429: 1–28.

[31] Hawlitschek O, Nagy ZT, Glaw F (2012) Island evolution and systematic revision of Comoran snakes: why and when subspecies still make sense. PLoS ONE 7(8): e42970. 22937005 10.1371/journal.pone.0042970 PMC3427315

[32] Hawlitschek O, Glaw F (2013) The complex colonization history of nocturnal geckos (*Paroedura*) in the Comoros Archipelago. Zool Scri 42: 135–150.

[33] Maddison WP, Maddison DR (2009) Mesquite: A modular system for evolutionary analysis. Version 2.72. Available via http://mesquiteproject.org.

[34] Katoh K, Misawa K, Kuma K, Miyata T (2002) MAFFT: a novel method for rapid multiple sequence alignment based on fast Fourier transform. Nucleic Acids Res 30: 3059–3066. 12136088 10.1093/nar/gkf436 PMC135756

[35] Katoh K, Toh H (2008) Improved accuracy of multiple ncRNA alignment by incorporating structural information into a MAFFT-based framework. BMC Bioinform 9: 212. 10.1186/1471-2105-9-212 PMC2387179 18439255

[36] Xia X, Xie Z, Salemi M, Chen L, Wang Y (2003) An index of substitution saturation and its application. Mol Phylogenet Evol 26: 1–7. 12470932 10.1016/s1055-7903(02)00326-3

[37] Xia X, Lemey P (2009) Assessing substitution saturation with DAMBE. In: Lemey P, Salemi M, Vandamme AM, eds. The Phylogenetic handbook: A practical approach to DNA and protein phylogeny, 2nd edition Cambridge University Press. pp 615–63.

[38] Xia X, Xie Z (2001) DAMBE: Data analysis in molecular biology and evolution. J Hered 92: 371–373. 11535656 10.1093/jhered/92.4.371

[39] Tamura K, Peterson D, Peterson N, Stecher G, Nei M, et al (2011) MEGA5: Molecular Evolutionary Genetics Analysis using maximum likelihood, evolutionary distance, and maximum parsimony methods. Mol Biol Evol 28: 2731–2739. 21546353 10.1093/molbev/msr121 PMC3203626

[40] Posada D (2008) jModelTest: Phylogenetic Model Averaging. Mol Biol Evol 25: 1253–1256. 18397919 10.1093/molbev/msn083

[41] Stamatakis A (2006) RAxML-VI-HPC: maximum likelihood-based phylogenetic analyses with thousands of taxa and mixed models. Bioinformatics 22: 2688–2690. 16928733 10.1093/bioinformatics/btl446

[42] Silvestro D, Michalak I (2011) raxmlGUI: a graphical front-end for RAxML. Org Divers Evol 12(4): 335–337.

[43] Ronquist F, Huelsenbeck JP (2003) MrBayes 3: Bayesian phylogenetic inference under mixed models. Bioinformatics 19: 1572–1574. 12912839 10.1093/bioinformatics/btg180

[44] Miller MA, Pfeiffer W, Schwartz T (2010) Creating the CIPRES Science Gateway for inference of large phylogenetic trees. In: Proceedings of the Gateway Computing Environments Workshop (GCE). 14 Nov. 2010, New Orleans, LA. pp 1–8.

[45] Rambaut A, Drummond A (2009) Tracer v1.5, Available from http://beast.bio.ed.ac.uk/Tracer.

[46] Meier R, Kwong S, Vaidya G, Ng PKL (2006) DNA barcoding and taxonomy in Diptera: a tale of high intraspecific variability and low identification success. Syst Biol 55: 715–728. 17060194 10.1080/10635150600969864

[47] Tänzler R, Sagata K, Surbakti S, Balke M, Riedel A (2012) DNA Barcoding for community ecology – how to tackle a hyperdiverse, mostly undescribed Melanesian fauna. PLoS ONE 7(1): e28832. 22253699 10.1371/journal.pone.0028832 PMC3258243

[48] Posada D (2004) Collapse: describing haplotypes from sequence alignments. Available from http://mac.softpedia.com/get/Math-Scientific/Posada-Collapse.shtml

[49] Horner P (2007) Systematics of the snake-eyed skinks, *Cryptoblepharus* Wiegmann (Reptilia: Squamata: Scincidae) – an Australian based review. The Beagle Suppl 3: 21–198.

[50] Ota H, Hikida T, Matsui M, Mori A, Wynn AH (1991) Morphological variation, karyotype and reproduction of the parthenogenetic blind snake, *Ramphotyphlops braminus*, from the insular region of East Asia and Saipan. Amphibia-Reptilia 12: 181–193.

[51] Hey J, Pinho C (2012) Population genetics and objectivity in species diagnosis. Evolution 66: 1413–1429. 22519781 10.1111/j.1558-5646.2011.01542.x PMC5607743

[52] Emerick CM, Duncan RA (1982) Age progressive volcanism in the Comoros archipelago, western Indian Ocean and implication for Somali plate tectonics. Earth Planet Sc Lett 60: 415–428.

[53] Raxworthy CJ, Ingram CM, Rabibisoa N, Pearson RG (2007) Applications of ecological niche modeling for species delimitation: a review and empirical evaluation using day geckos (*Phelsuma*) from Madagascar. Syst Biol 56: 907–923. 18066927 10.1080/10635150701775111

[54] Hawlitschek O (2008) Reptiles and amphibians of the Comoro islands. Diploma thesis, University of Munich, 257 pp.

